# Impaired peripheral perfusion during anesthesia is associated with postoperative delirium in older patients undergoing minimally invasive colorectal surgery: a retrospective observational study

**DOI:** 10.3389/fmed.2026.1853233

**Published:** 2026-08-10

**Authors:** Yu Matsumoto, Shohei Nakatani, Tomomi Yamada, Takeshi Yoshida

**Affiliations:** 1 Department of Anesthesiology and Intensive Care Medicine, Osaka University Graduate School of Medicine, Suita, Osaka, Japan; 2 The Department of Medical Innovation Data Coordinating Center, Osaka University Hospital, Suita, Osaka, Japan

**Keywords:** cerebral hypoperfusion, ICDSC, perfusion index, peripheral perfusion, postoperative delirium, pulse oximetry

## Abstract

**Introduction:**

Postoperative delirium (POD) is a frequent postoperative complication in older patients and is associated with poor outcomes. Mean arterial pressure may not adequately reflect microcirculatory perfusion during anesthesia, and the perfusion index (PI), derived from pulse oximetry, is a promising noninvasive surrogate. This study aimed to investigate the association of intraoperative PI with POD in older adults undergoing minimally invasive colorectal surgery.

**Methods:**

This retrospective observational study included patients aged ≥65 years who underwent elective laparoscopic or robot-assisted colorectal surgery between July 2024 and July 2025. POD was assessed using the Intensive Care Delirium Screening Checklist twice daily for the first three postoperative days and defined as a score of ≥4. PI values were recorded every 3 s during anesthesia, and the mean PI was calculated for each patient. Predictive performance was evaluated using receiver operating characteristic analysis. Separate two-variable logistic regression models based on mean PI and one covariate were developed.

**Results:**

Ninety-eight patients (11 had POD) were included. The mean PI was lower for those with than for those without POD (median 1.5 [1.3–1.8] vs. 2.3 [1.7–3.1]; *P* = 0.0069). The mean PI showed moderate predictive value for POD (AUC, 0.75; 95% CI 0.61–0.89). The optimal cut-off value was 1.83, yielding 82% sensitivity and 70% specificity. The mean PI remained significantly associated with POD after adjusting for serum albumin and hemoglobin concentrations.

**Conclusions:**

Lower intraoperative mean PI was associated with POD and demonstrated moderate predictive ability. PI may complement conventional monitoring for perioperative POD risk assessment. Future studies are required to determine whether PI-guided intraoperative management can improve postoperative outcomes.

## 1 Background

Postoperative delirium (POD) is one of the most common postoperative complications in older patients undergoing surgery and is associated with increased morbidity, prolonged hospitalization, institutionalization, and long-term mortality (1–3). Therefore, identifying the predictors of the development of POD is important for its prevention and early treatment.

POD is a multifactorial complication, and one of its proposed mechanisms is cerebral hypoperfusion, which leads to impaired cerebral oxygen delivery (4). While conventional intraoperative circulatory monitoring is largely based on the mean arterial pressure (MAP), the macrocirculatory parameter MAP may be dissociated from the microcirculation and may not adequately guide circulatory optimization during anesthesia because of perioperative sympathetic or medically induced vasoconstriction (5). Several clinical studies have demonstrated that normalization of macrocirculatory parameters does not necessarily ensure the restoration of microcirculatory perfusion (6, 7). Assuming that peripheral blood flow is reduced to augment central blood volume and perfusion of vital organs during hemodynamic deterioration (8), a noninvasive method for detecting impaired peripheral perfusion may be relevant for identifying circulatory instability as demonstrated during septic and cardiogenic shock (9). An association between intraoperative MAP and POD has not been consistently demonstrated, and MAP-based strategies for POD prevention have not been established (10–12). Therefore, MAP may not adequately capture cerebral hypoperfusion relevant to POD development.

What are novel approaches for assessing microcirculation? Near-infrared spectroscopy (NIRS) is promising for non-invasively evaluating the oxygen supply/demand balance in frontal brain tissue and providing real-time regional cerebral oxygen saturation (rSO_2_). Intraoperative reduction of rSO_2_ may indicate a clinically relevant association with cognitive dysfunction (13, 14). However, this conclusion remains controversial. The association between rSO_2_ and POD has not been consistently demonstrated (15), and some recent randomized-controlled trials have demonstrated that NIRS-guided intervention has no effect on neurocognitive disorders (16, 17). Moreover, cerebral NIRS monitoring is limited to cardiac surgeries and carotid endarterectomies. Thus, simpler and more widely available peripheral perfusion–related parameters are needed for POD risk assessment during anesthesia.

The perfusion index (PI), which is obtained from pulse oximetry, represents the ratio of pulsatile to non-pulsatile blood flow obtained from the plethysmographic waveform. It has been proposed as a noninvasive surrogate marker of peripheral microcirculatory perfusion, reflecting peripheral blood flow and vascular tone (18, 19). PI can be continuously monitored without additional equipment or operator dependency, and it may serve as a practical indicator of hemodynamic instability and impaired tissue perfusion during surgery. However, the association between intraoperative PI and POD has not been well characterized. Based on this, we hypothesized that lower intraoperative PI values would be associated with the development of postoperative delirium.

The aim of this study was to investigate the association between intraoperative PI and POD in older patients undergoing laparoscopic or robot-assisted colorectal surgery. PI may offer a simple and widely available marker for perioperative POD risk stratification and inform future PI-guided preventive strategies if validated.

## 2 Methods

### 2.1 Study design and ethical approval

This retrospective observational study was conducted at the Osaka University Hospital. The institutional review board approved the study protocol, and waived the requirement for written informed consent in accordance with the institutional opt-out policy.

### 2.2 Study population

Consecutive patients aged 65 years or older who underwent elective laparoscopic or robot-assisted colorectal surgery between July 2024 and July 2025 were screened for inclusion. The exclusion criteria included conversion to open surgery, an American Society of Anesthesiologists physical status classification of ≥3, and surgery performed for inflammatory bowel disease.

### 2.3 Assessment of postoperative delirium

POD was assessed using the Intensive Care Delirium Screening Checklist (ICDSC). ICDSC scores were routinely recorded in the ICU and/or general ward twice daily for the first three postoperative days. The ICDSC scores were obtained from electronic medical records. Patients were eligible for inclusion only if at least four of the six scheduled ICDSC assessments had been completed, to ensure adequate assessment of postoperative delirium. No patients were excluded because of incomplete ICDSC assessments. POD was defined as an ICDSC score of 4 or higher at any time during the observation period.

### 2.4 Perfusion index measurement

The intraoperative PI data were obtained from the pulse oximetry signal recorded by the anesthesia monitoring system using a disposable pulse oximeter probe (TL-271T; Nihon Kohden, Tokyo, Japan). The probe was attached to a finger for all the patients, and PI values were automatically recorded at 3-s intervals. All numerically available PI values were extracted from the start to the end of anesthesia, and the mean PI value for each patient was calculated from them. Intraoperative management was performed according to routine clinical practice. Body temperature was routinely monitored using either a bladder or a pharyngeal temperature probe and maintained using standard warming measures throughout surgery. Bispectral index monitoring was routinely used during total intravenous anesthesia, whereas its use during inhalational anesthesia was left to the discretion of the attending anesthesiologist. Fluid therapy and vasopressor administration were adjusted according to the patient's intraoperative hemodynamic status.

### 2.5 Outcomes

The primary outcome was the association between the intraoperative mean perfusion index and POD. The secondary outcomes included the discriminative performance of the mean PI for predicting postoperative delirium, which was evaluated using receiver operating characteristic (ROC) curve analysis. Discrimination was quantified using the area under the curve (AUC), and the optimal cutoff value was determined.

### 2.6 Impact of vasopressor administration on PI

As a supplementary exploratory analysis, the effect of vasopressor administration on PI was evaluated. To isolate the effect of each vasopressor, we randomly selected bolus events of either ephedrine (4 mg) or phenylephrine (50 μg) administered during anesthesia. Phenylephrine 50 μg and ephedrine 4 mg are commonly administered as approximately equipotent bolus doses for the treatment of hypotension (20). For each selected case, PI values during the 3 min immediately before and after bolus administration were extracted from the anesthesia monitoring system. The mean pre- and post-administration PI values were calculated. For each vasopressor group, the pre- and post-administration PI values were compared using the Wilcoxon signed-rank test. In addition, the change in PI (ΔPI), defined as the post-administration mean PI minus the pre-administration mean PI, was calculated for each patient and compared for the phenylephrine and ephedrine groups using the Mann–Whitney *U*-test.

### 2.7 Statistical analysis

Continuous variables are summarized as medians with interquartile ranges, whereas categorical variables are summarized as numbers and percentages. Patients with and without postoperative delirium were compared using the Mann–Whitney *U*-test for continuous variables and Fisher's exact test for categorical variables, as appropriate. ROC curves were constructed, and the area under the curve (AUC) with a 95% confidence interval (CI) was calculated. The optimal cutoff value was determined using the Youden index. Given the limited number of POD events, we developed separate two-variable logistic regression models including mean PI and one covariate (serum albumin concentration, hemoglobin concentration, or duration of surgery) to minimize overfitting. These covariates were selected because they are established or clinically plausible risk factors for POD and were objectively and reliably available in the electronic medical records, allowing consistent extraction in this retrospective study (21). POD was assessed using the ICDSC by bedside nurses as part of routine clinical care, whereas PI values were extracted separately from the anesthesia records in this study. This ensured that outcome assessment and PI data collection were performed independently. No missing data were present for the variables included in the statistical analyses; therefore, no imputation or other missing-data handling procedures were required.

All statistical analyses were performed using EZR software. Figures were generated using GraphPad Prism (version 10.4.2; GraphPad Software, San Diego, CA, USA) and EZR software.

All statistical tests were two-sided, and *P* values of < 0.05 were considered statistically significant.

## 3 Results

**Figure 1:**
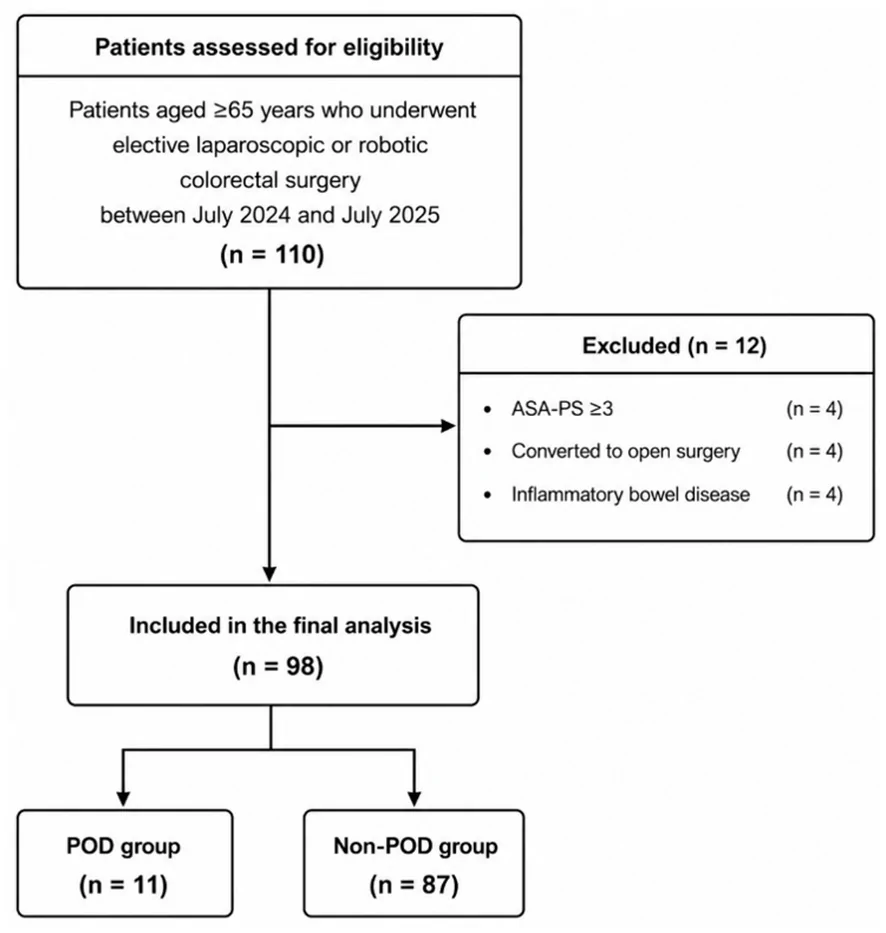
Flow diagram of patient selection. Patients aged ≥65 years who underwent elective laparoscopic or robotic colorectal surgery between July 2024 and July 2025 were screened for eligibility. After exclusion of patients with an American Society of Anesthesiologists Physical Status (ASA-PS) classification ≥3, conversion to open surgery, or inflammatory bowel disease, 98 patients were included in the final analysis. All included patients had complete intraoperative perfusion index (PI) data.

Ninety-eight patients, comprising 11 with POD and 87 without POD, were included in the analysis (Figure 1). The baseline patient characteristics and intraoperative variables are summarized in Table 1. Several baseline and intraoperative variables, including albumin concentration, hemoglobin concentration, and duration of surgery, differed between the groups.

**Table 1:** Baseline characteristics and intraoperative variables for the patients with and without postoperative delirium.

Variables	POD (*n* = 11)	No POD (*n* = 87)	*P* value
Female gender, *n* (%)	5 (45)	46 (53)	0.753
Age (year)	78 (76.5–85.3)	75 (72–79)	0.0928
BMI (kg/m^2^)	22.6 (21.4–25.1)	22.6 (20.2–25.5)	0.928
Alb (g/dL)	3.6 (3.1–4.0)	4.1 (3.8–4.4)	0.012
Hb (g/dL)	11.1 (10.4–11.8)	12.4 (11.1–13.7)	0.0315
Comorbidity, *n* (%)			
Hypertension	5 (46)	39 (45)	1.00
Diabetes mellitus	2 (18)	18 (21)	1.00
History of stroke	2 (18)	6 (7)	0.22
History of dementia	0 (0)	1 (1)	1.00
History of myocardial infarction	1 (9)	6 (7)	0.578
Atrial fibrillation	0 (0)	3 (3)	1.00
Surgical approach			
Robotic	7 (64)	55 (63)	1.00
Laparoscopic	4 (36)	32 (37)	
Surgical site			
Colon	7 (64)	56 (64)	1.00
Rectum	4 (36)	31 (36)	
Operative variables			
Duration of surgery (min)	327 (235–556)	253 (185–297.2)	0.029
Volatile anesthetics	8 (73)	76 (87)	0.19
Total intravenous anesthesia	3 (27)	11 (13)	

Data are presented as median (interquartile range) or number (%), as appropriate. Comparisons of groups were performed using the Mann–Whitney *U*-test for continuous variables and Fisher's exact test for categorical variables. Statistical significance was defined as a two-sided a two-sided *P* values of < 0.05. Alb, serum albumin; BMI, body mass index; Hb, hemoglobin; POD, postoperative delirium.

### 3.1 Primary outcomes

**Figure 2:**
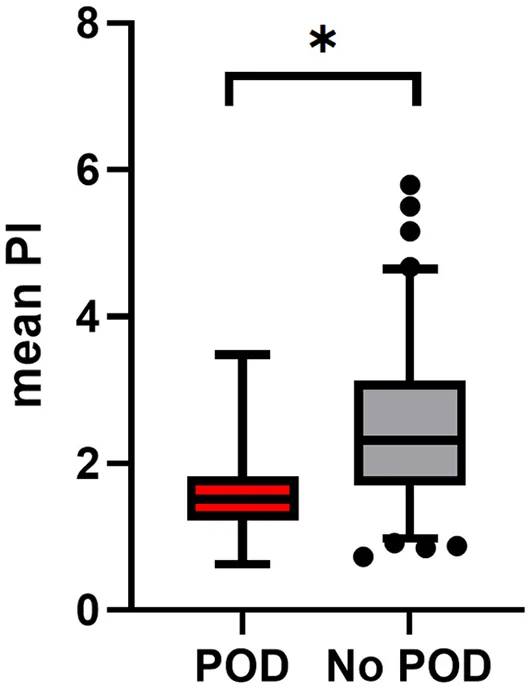
Intraoperative mean perfusion indexes of patients with and without postoperative delirium. Box-and-whisker plots show the intraoperative mean perfusion index (PI) for patients with (*n* = 11) and without (*n* = 87) POD. Boxes represent the interquartile range, the center line indicates the median, and whiskers indicate the 5th−95th percentiles. The dots represent outliers. The mean PI was significantly lower for the POD group than for the non-POD group (*P = 0.0069; Mann–Whitney *U*-test). PI, perfusion index; POD, postoperative delirium.

The overall median intraoperative mean PI was 2.2 (1.5–3.0). It was significantly lower for patients who developed POD than for those who did not (1.5 [1.3–1.8] vs. 2.3 [1.7–3.1], respectively; *P* = 0.0069; Figure 2).

### 3.2 Secondary outcomes

**Figure 3:**
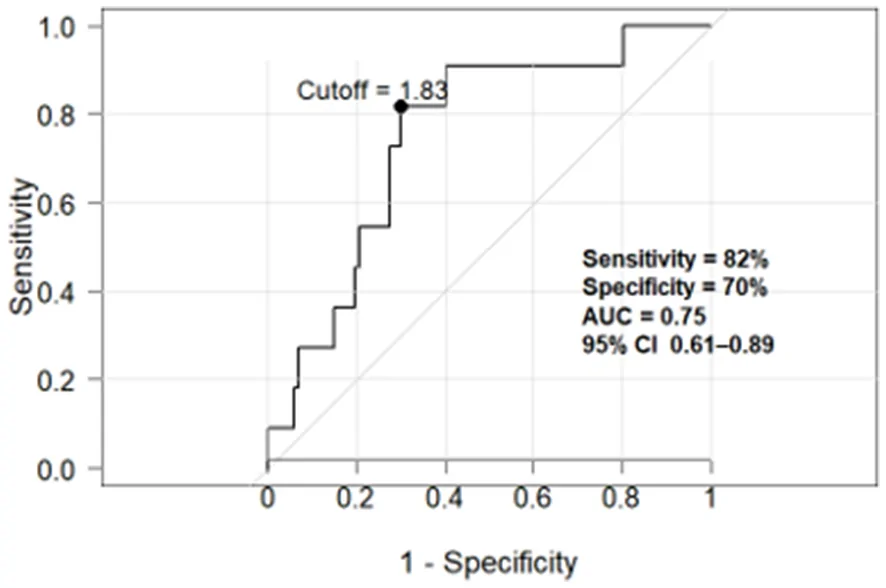
Receiver operating characteristic curve of intraoperative mean perfusion index for predicting postoperative delirium. ROC curve evaluating the discriminative performance of the intraoperative mean PI for predicting POD (*n* = 98; POD, *n* = 11; no POD, *n* = 87). The AUC was 0.75 (95% confidence interval, 0.61–0.89). The optimal cutoff value determined using the Youden index was 1.83, yielding a sensitivity of 82% and a specificity of 70%. AUC, area under the curve; PI, perfusion index; POD, postoperative delirium; ROC, receiver operating characteristic.

ROC curve analysis demonstrated that mean PI had moderate discriminative ability for predicting POD, with an area under the curve (AUC) of 0.75 (95% confidence interval [CI], 0.61–0.89). Using the Youden index, the optimal cut-off value of the mean PI was determined to be 1.83, yielding a sensitivity of 82% and a specificity of 70% for predicting POD (Figure 3).

Given the limited number of POD events, we developed separate two-variable logistic regression models, including the mean PI and one covariate (albumin concentration, hemoglobin concentration, or duration of surgery). The model adjusted for serum albumin concentration (Model 1) revealed a significant association of POD with mean PI (odds ratio [OR], 0.373; 95% confidence interval [CI], 0.145–0.962; *P* = 0.041), but a borderline association with serum albumin concentration (OR, 0.334; 95% CI, 0.110–1.010; *P* = 0.052). Similarly, the model adjusted for hemoglobin concentration (Model 2) revealed a significant association of POD with mean PI (OR, 0.337; 95% CI, 0.133–0.855; *P* = 0.022), but not with hemoglobin concentration (OR, 0.734; 95% CI, 0.515–1.050; *P* = 0.087).

The model adjusted for duration of surgery (Model 3) revealed a significant association of POD with the duration of surgery (OR, 1.010; 95% CI, 1.000–1.010; *P* = 0.027) but not with mean PI (OR, 0.385; 95% CI, 0.145–1.020; *P* = 0.056) (Table 2).

**Table 2:** Two-variable logistic regression models for postoperative delirium.

Variable	Model1 (PI + Alb)		Model2 (PI + Hb)		Model3 (PI + Op duration)	
	OR (95%CI)	*P* value	OR (95%CI)	*P* value	OR (95%CI)	*P* value
Mean PI	0.373 (0.145–0.962)	0.041	0.337 (0.133–0.855)	0.022	0.385 (0.145–1.020)	0.056
Alb (g/dL)	0.334 (0.110–1.010)	0.052				
Hb (g/dL)			0.734 (0.515–1.050)	0.087		
Duration of surgery (min)			1.010 (1.000–1.010)	0.027		

Values are odds ratios (ORs) with 95% confidence intervals (CIs). Due to the limited number of postoperative delirium events, separate two-variable models based on the mean perfusion index and one covariate, were developed to minimize overfitting.

Alb, serum albumin; Hb, hemoglobin; PI, perfusion index.

### 3.3 Supplementary data

PI responses to vasopressor bolus administration were evaluated for seven randomly selected patients who accounted for 20 bolus events (phenylephrine, *n* = 10; ephedrine, *n* = 10). The change in PI (ΔPI) differed significantly after administering the agents (ΔPI for phenylephrine, −0.33 [−0.88 to −0.13] vs. ΔPI for ephedrine, 0.05 [−0.04 to 0.36]; *P* = 0.0007). PI decreased significantly after administering phenylephrine (pre 3.08 vs. post 2.33; *P* = 0.002), but no significant change was observed after administering ephedrine (pre 2.9 vs. post 2.9; *P* = 0.275; Supplementary Table 1).

## 4 Discussion

In this study, we demonstrated that a lower intraoperative mean PI was significantly associated with POD in older patients undergoing elective minimally invasive colorectal surgery. Patients who developed POD had persistently reduced PI values throughout anesthesia, and the mean PI demonstrated a moderate discriminative ability for POD prediction. These findings suggest that pulse oximetry–derived PI may provide clinically relevant information for POD risk assessment during anesthesia.

PI is easy to measure and can be continuously displayed on a monitor. A growing number of clinical studies support the use of PI in various clinical scenarios as an indicator of outcomes and organ function (19). Su et al. found that PI values below 1.37 during the first 24 h after ICU admission were a good predictor of in-ICU mortality (22). Another study has shown that PI values below 1.4 on the second day after surgery is predictive of severe postoperative complications independent of systemic hemodynamics (23). Perfusion index has been extensively studied in patients with critical illness and postoperative patients, but evidence on intraoperative PI values remains limited. In this study, the overall median intraoperative mean PI was 2.2, which was lower than that reported in a previous study of patients under general anesthesia (mean PI, 3.9) (24). This finding may reflect the specific features of our cohort—older patients undergoing laparoscopic or robot-assisted colorectal surgery—because both advanced age and minimally invasive surgical conditions (such as pneumoperitoneum and patient positioning) are known to alter peripheral vascular tone and perfusion (25–27).

To focus on a population at higher risk of POD, we limited our analysis to patients aged 65 years or older (28). The incidence of POD in the present cohort was 11%, which is comparable to that reported in previous studies of older patients undergoing minimally invasive colorectal surgery (29). In addition, patients who developed POD in this study had lower hemoglobin and serum albumin concentrations and longer operative durations, all of which are established risk factors (21, 30–32). These findings suggest that our study population appropriately represents a typical older adult surgical cohort at risk of POD and supports the external validity of our results.

For our patient sample, mean PI was significantly lower in patients with POD, and ROC analysis demonstrated moderate predictive accuracy (AUC 0.75). In the present study, the optimal mean PI cut-off value for predicting POD was 1.83. Prior intraoperative studies have shown that PI values < 1.8 reliably reflect impaired peripheral perfusion under general anesthesia, as indicated by prolonged capillary refill time and high discriminative accuracy (AUC 0.99) (33). Our study-derived cutoff value should be interpreted cautiously, but its agreement with previously reported thresholds supports its physiological plausibility. In our supplementary analysis, mean intraoperative MAP was not significantly associated with POD (Supplementary Figure 1). This finding suggests that PI may capture aspects of peripheral or microcirculatory perfusion that are not adequately reflected by conventional blood pressure–based indices, potentially enabling the detection of previously unrecognized perfusion-related risk for POD.

Multivariate analysis was performed to determine whether PI could serve as an independent predictor of POD. The number of POD events was limited, and we restricted the number of covariates in each model. The mean PI remained significantly associated with POD after adjusting for serum albumin or hemoglobin concentration. This suggests that PI provides information beyond nutritional status and oxygen-carrying capacity, both of which have been reported as risk factors for POD. In contrast, the mean PI was not statistically significant when the duration of surgery was included in the model. The duration of surgery has been considered a perioperative factor associated with POD risk after colorectal surgery and may also act as a proxy for surgical burden (34). Prolonged surgery may reflect an increased surgical burden, which may contribute to impaired peripheral perfusion during the perioperative period (35). Therefore, both low intraoperative PI and prolonged surgery may capture overlapping information related to surgical burden and its effects on peripheral perfusion. This overlap may explain why the association between PI and POD was attenuated after adjusting for the duration of surgery. Alternatively, duration of surgery may partially increase the risk of POD by worsening peripheral perfusion. In this case, adjusting for operative duration may remove part of the perfusion-related effect captured by the PI and could lead to overadjustment. In addition, the limited statistical power due to the small number of POD events cannot be excluded. These issues warrant further investigation in a larger cohort.

Our findings suggest that PI-guided hemodynamic management is a promising strategy for POD prevention. The choice of vasopressor is an important determinant of peripheral and microcirculatory perfusion. Previous studies have reported a higher incidence of POD in patients receiving phenylephrine than in those receiving ephedrine, despite similar blood pressure control (36). Phenylephrine increases vascular resistance through α-adrenergic stimulation, whereas ephedrine primarily augments cardiac output (37). Consistent with these observations, our supplementary analysis demonstrated a significant decrease in the PI following phenylephrine administration, whereas no significant change in the PI was observed after ephedrine administration (Supplementary Figure 2). These findings suggest that PI may capture clinically relevant perfusion differences that are not reflected by arterial blood pressure alone. Prospective interventional studies are required to determine whether PI-guided vasopressor selection can reduce the incidence of POD and improve postoperative neurocognitive outcomes.

This study had some limitations. First, it was a retrospective single-center observational study that included all eligible consecutive patients during the study period. Therefore, no formal sample size calculation was performed, and causality could not be inferred. In addition, perioperative management, including the choice of surgical approach and intraoperative anesthetic management such as vasopressor selection, was not standardized because these decisions were made according to routine clinical practice. Consequently, selection bias, residual confounding, and the possibility of underpowered analyses cannot be excluded. The present findings should be considered exploratory, and their generalizability to other surgical populations or institutions remains uncertain. Second, the number of POD events was small, limiting the statistical power and the extent of multivariable adjustment. We had to use separate two-variable models to reduce overfitting. Residual confounding from unmeasured perioperative factors cannot be excluded. In addition, because of the limited number of POD events, additional assessments of model performance, such as calibration analysis, goodness-of-fit testing, and internal validation, were not considered reliable in this exploratory analysis. Third, the PI is influenced by multiple perioperative factors (such as anesthetic depth, temperature, sympathetic tone, and vasoactive drug use), which could not be fully standardized in this retrospective study. Thus, PI should be regarded as an integrated marker of peripheral circulatory status during anesthesia. Importantly, PI should not be interpreted as a direct measure of cerebral perfusion. Although persistently lower intraoperative PI was associated with an increased risk of POD in the present study, it remains unknown whether PI-guided intraoperative management can reduce the incidence of POD. Future prospective interventional studies are warranted to determine the clinical utility of PI-guided management for POD prevention. Fourth, POD was assessed using the ICDSC twice daily for three postoperative days, and some episodes of delirium may have been missed because of its fluctuation over time. This likely underestimated the true association between PI and POD. Finally, the PI cut-off value (1.83) was derived from a single-center cohort with a specific population, and this threshold should be regarded as hypothesis-generating. External validation in larger, independent multicenter cohorts is required before its routine clinical application.

## 5 Conclusions

In older patients undergoing minimally invasive colorectal surgery, a lower intraoperative mean PI was associated with postoperative delirium and demonstrated moderate predictive ability. PI may provide complementary information beyond conventional blood pressure monitoring and warrants further investigation as a perfusion-related marker for assessing POD risk.

## Data Availability

The data analyzed in this study is subject to the following licenses/restrictions. The datasets generated and/or analyzed during the current study are not publicly available due to patient privacy and institutional restrictions but are available from the corresponding author on reasonable request. Requests to access these datasets should be directed to Yu Matsumoto, matsumoto.yuu.e2w@osaka-u.ac.jp.

## Supplementary material

SUPPLEMENTARY FIGURE 1: Intraoperative mean MAP of patients with and without postoperative delirium. The data of 44 patients with invasive arterial catheters were included in the analysis (POD, *n* = 10; no POD, *n* = 34). For each patient, the mean MAP during anesthesia was calculated. The values were compared between groups. The median mean MAP was 72.6 mmHg for the POD group and 75.3 mmHg for the no POD group. No statistically significant differences were observed between the groups (Mann–Whitney *U*-test, *P* = 0.17). Boxes represent the interquartile range, the center line indicates the median, and whiskers indicate the 5th−95th percentiles. The dots represent outliers. MAP, mean arterial pressure; POD, postoperative delirium.

SUPPLEMENTARY FIGURE 2: Effects of phenylephrine and ephedrine bolus administration on perfusion index. (a) Changes in PI before and after bolus administration of phenylephrine (50 μg) or ephedrine (4 mg). PI values represent the mean PI during the 3 min immediately before (Pre) and after (Post) each bolus event. For each vasopressor group, pre- and post-administration PI values were compared using the Wilcoxon signed-rank test. Following phenylephrine administration, PI was significantly lower in the Post period compared with the Pre period (^*^*P* = 0.002), whereas no significant change was observed after ephedrine administration (*P* = 0.275). (b) Changes in PI (ΔPI), defined as post-administration mean PI minus pre-administration mean PI, after phenylephrine and ephedrine bolus events. ΔPI was positive after ephedrine administration and negative after phenylephrine administration (ΔPI: 0.05 [−0.04 to 0.36] vs. −0.33 [−0.88 to −0.13], respectively; Mann–Whitney *U*-test, ^**^*P* = 0.0007). Seven patients accounted for 20 bolus events (phenylephrine, *n* = 10; ephedrine, *n* = 10). Data are shown as median with interquartile range. PI, perfusion index; ΔPI, change in perfusion index.
